# *A priori* prediction of breast tumour response to chemotherapy using quantitative ultrasound imaging and artificial neural networks

**DOI:** 10.18632/oncotarget.26996

**Published:** 2019-06-11

**Authors:** Hadi Tadayyon, Mehrdad Gangeh, Lakshmanan Sannachi, Maureen Trudeau, Kathleen Pritchard, Sonal Ghandi, Andrea Eisen, Nicole Look-Hong, Claire Holloway, Frances Wright, Eileen Rakovitch, Danny Vesprini, William Tyler Tran, Belinda Curpen, Gregory Czarnota

**Affiliations:** ^1^ Physical Sciences, Sunnybrook Research Institute, Sunnybrook Health Sciences Centre, Toronto, ON, Canada; ^2^ Department of Medical Biophysics, Faculty of Medicine, University of Toronto, Toronto, ON, Canada; ^3^ Division of Medical Oncology, Department of Medicine, Sunnybrook Health Sciences Centre, Toronto, ON, Canada; ^4^ Surgical Oncology, Department of Surgery, Sunnybrook Health Sciences Centre, Toronto, ON, Canada; ^5^ Department of Radiation Oncology, Odette Cancer Centre, Sunnybrook Health Sciences Centre, Toronto, ON, Canada; ^6^ Department of Radiation Oncology, Faculty of Medicine, University of Toronto, Toronto, ON, Canada; ^7^ Department of Medical Imaging, Sunnybrook Health Sciences Centre, and Faculty of Medicine, University of Toronto, Toronto, ON, Canada

**Keywords:** quantitative ultrasound, artificial neural networks, ultrasound spectroscopy, tumour response assessment, prognostic biomarker

## Abstract

We demonstrate the clinical utility of combining quantitative ultrasound (QUS) imaging of the breast with an artificial neural network (ANN) classifier to predict the response of breast cancer patients to neoadjuvant chemotherapy (NAC) administration prior to the start of treatment.

Using a 6 MHz ultrasound system, radiofrequency (RF) ultrasound data were acquired from 100 patients with biopsy-confirmed locally advanced breast cancer prior to the start of NAC. Quantitative ultrasound mean parameter intensity and texture features were computed from the tumour core and margin, and were compared to the clinical/pathological response and 5-year recurrence-free survival (RFS) of patients. A multi-parametric QUS model in conjunction with an ANN classifier predicted patient response with 96 ± 6% accuracy, and a 0.96 ± 0.08 area under the receiver operating characteristic curve (AUC), compared to 65 ± 10 % accuracy and 0.67 ± 0.14 AUC achieved using a K-Nearest Neighbour (KNN) algorithm. A separate ANN model predicted patient RFS with 85 ± 7% accuracy, and a 0.89 ± 0.11 AUC, whereas the KNN methodology achieved a 58 ± 6 % accuracy and a 0.64 ± 0.09 AUC.

The application of ANN for classifying patient response based on tumour QUS features performs well in terms of predicting response to chemotherapy. The findings here provide a framework for developing personalized *a priori* chemotherapy selection for patients that are candidates for NAC, potentially resulting in improved patient treatment outcomes and prognosis.

## INTRODUCTION

Neoadjuvant chemotherapy (NAC) is the primary up-front treatment modality for patients with locally advanced breast cancer (LABC). This aggressive form of cancer typically presents with tumours larger than 5 cm and extensive nodal involvement. Since the tumours are often inoperable, the goal of NAC is to reduce tumour volume. NAC may also be given to facilitate breast conserving surgery for patients who would otherwise require a mastectomy. There is a strong correlation between a pathological complete response (pCR) to NAC and cancer-free survival. Despite the availability of a wide spectrum of systemic and targeted drugs, due to genetic and epigenetic factors, most patients do not achieve pathologic complete response to NAC. Response is typically determined at the end of several months of treatment. In this light, there is growing interest in the discovery of biomarkers to predict therapy response with the aim of optimizing treatment reducing morbidity by avoiding futile treatments, and improving prognosis. For instance, diffusion-weighted MRI (DW-MRI) has been demonstrated to predict clinical response of breast tumours as early as after one cycle of chemotherapy [1]. Positron emission tomography (PET) imaging of breast cancer patients using a fluorodeoxyglucose (FDG) contrast agent has detected response-related changes in the tumour after one cycle of chemotherapy [2]. Additionally, diffuse optical imaging (DOI) studies of breast cancer have measured a significant increase in haemoglobin concentration, water content, and tissue optical index in responding patients as early as one week after the start of chemotherapy [3].

To date, the majority of studies have focused on biomarkers reflective of treatment-induced changes in functional and/or structural properties of the tumour (i.e. monitoring biomarkers). However, there is a growing shift of attention toward biomarkers reflecting inherent tumour biology (i.e. predictive biomarkers), which do not require any treatment to be administered. From a non-imaging perspective, pre-treatment levels of immunohistochemical markers, including Ki-67, HER2, and circulating nucleosomes have been linked to the likelihood of breast tumour’s response to NAC [4–7]. From an imaging perspective, a growing body of research exists in the area of pre-treatment imaging biomarkers. In a recent study, diffuse optical spectroscopic (DOS) imaging of LABC patients indicated that patients with a pathologically complete response have significantly higher up-front haemoglobin concentration levels than those with pathologically incomplete response with *p* = 0.01, AUC =1.0 [8]. A more recent DOS study demonstrated that changes in tissue optical index and baseline oxygen saturation levels are indicators of pCR with an AUC of 0.83 [9]. Intra-tumoural and peri-tumoural radiomic features of dynamic contrast-enhanced MRI (DCE-MRI) of the breast have been demonstrated to be predictive of pCR prior to treatment with AUC of 0.74 [10]. In the area of PET imaging, the median progression-free survival of patients with estrogen receptor (ERe)- positive, human epithelial growth factor 2 (HER2)- negative breast tumours undergoing endocrine therapy was linked to their FDG uptake prior to treatment [11].

The use of clinical ultrasound has been established in the field of medical imaging as a cost-effective modality with high penetration depth (~7 cm) and real-time imaging capability. Furthermore, the raw ultrasound radiofrequency (RF) backscatter signal contains information about tissue microstructure, which is not resolvable in conventional ultrasound images (B-mode images). Quantitative ultrasound (QUS) techniques examine the frequency dependence of the RF signal backscattered from tissues and have been applied *in vivo* in a variety of applications to reveal information about tissue microstructure, enabling the differentiation of disease from normal tissue and the characterization of disease into its subtypes. For instance, parameters derived from the linear regression analysis of the RF power spectrum, including midband fit (MBF), spectral slope (SS), and spectral 0-MHz intercept (SI), have been used to characterize intraocular tumours and to detect prostate cancer, cardiovascular disease, and cancerous lymph nodes [12–15].

Broader frequency bandwidths further permit the estimation of advanced parameters such as average (effective) scatterer diameter (ASD) and average (effective) acoustic concentration (AAC), which are derived by fitting a scattering model to the RF data [16]. These parameters have effectively differentiated mouse carcinomas from rat fibroadenomas [17] and have demonstrated potential for use in breast tumour grading [18, 19] and diagnosis [20]. Recent pre-clinical studies have determined, using both high frequency (>20 MHz) and clinical frequency (<10MHz) ranges of ultrasound, that QUS can be used to detect and quantify tumour cell death *in vivo* in response to various treatments including photodynamic therapy, radiation therapy, chemotherapy, and anti-vascular therapy [21–24]. Furthermore, a recent pilot clinical study [25] demonstrated the effectiveness of using textural features extracted from QUS spectral images (MBF and SI) to detect breast tumour responses to neoadjuvant chemotherapy as early as one week into several-month-long chemotherapy treatments. The mean of intensity of ASD and AAC images derived from ultrasound backscatter data have also been effective at detecting treatment response in a similar clinical application [26].

Scatterer spacing, also known as spacing among scatterers (SAS), has also been investigated as a tissue characterization biomarker for tissues containing detectable periodicity in their structural organization. Previous studies have investigated the potential of SAS mainly for characterizing diffuse diseases of the liver [27–30]. For instance, in [28], the inter-scatterer-distribution (ISS) and the mean scatterer spacing (MSS) have been investigated for characterizing focal diseases of the liver using wavelet transform-based methods [28]. The MSS was considered for characterization of pathological human liver using Fourier transform-based methods [29]. The terms SAS and MSS are used interchangeably in the literature to refer to the mean scatterer spacing. More recently, SAS demonstrated discriminative power in breast tumour grading and therapy response applications [19, 31]. Motivated by those studies, we recently investigated whether pre-treatment values of QUS biomarkers can differentiate between therapy responsive and non-responsive tumours [32]. A multiparametric QUS model was developed using two regions of interest (ROIs) – the tumour core and a 5 mm margin of surrounding tissue. For each ROI, mean of intensity and texture features of QUS images were computed and incorporated into a k-nearest neighbour (KNN) classifier. Results from 56 LABC patients, indicated a response prediction accuracy of 88%, which was linked to a 5-year recurrence-free survival (RFS). However, as data becomes more complex (i.e. as data dimensionality increases), KNN performance typically deteriorates. In classification problems with high dimensionality, such as this study, an artificial neural network (ANN) classifier is a suitable choice [33]. An ANN is a nonlinear classifier that learns patterns in a data set using an interconnected network of “neurons” (elements with multiple inputs and one output) with a predefined activation rule. In the present study a previously examined cohort [32] was expanded from 56 patients to 100 patients. The data set was balanced prior to supervised learning and a more advanced model – an ANN model - was trained to predict the response and 5-year RFS of LABC patients undergoing NAC. The results were then compared with those obtained from the previously used KNN model. In addition to the conventional binary response classification (response versus non-response) done previously, a three-class grouping scheme was also investigated here. This included complete, partial, and non-response classification. Finally, whereas previous work reported Kaplan-Meier 5-year RFS curves of responding and non-responding patients, here, the 5-year RFS of patients was separately predicted using QUS-based biomarkers directly.

## RESULTS

### Patient clinical characteristics

Table 1 presents a statistical summary of patient clinical characteristics including age, tumour size, estrogen receptor (ERe) status, progesterone receptor (PRe) status, and human epithelial growth factor 2 (HER2) status. The patients are separated according to response groups. Based on the modified response (MR) scoring system and binary classification of response described previously, of the 100 patients in the study, 83 patients responded to treatment and 17 patients did not respond to treatment. Responders had a mean age of 50 ± 10 years and non-responders had a mean age of 47 ± 12 years. Responders and non-responders had similar mean initial tumour sizes of 5.6 ± 2.7 cm and 5.9 ± 2.8 cm, respectively. The proportion of patients with ERe, PRe, and HER2 positive tumours in responder and non-responder groups are presented in Table 1. Statistical analysis using a chi-square test of independence demonstrated a significant correlation between complete response and HER2-postivitiy (*p* = 0.002), whereas no statistically significant correlation was found between response and any of the hormone-based markers. In terms of histological subtype, the majority of the patients in both groups were diagnosed with invasive ductal carcinoma (91 % and 94 % in responder and non-responder groups, respectively), with a small number of other subtypes such as invasive lobular carcinoma and invasive mammary carcinoma, as presented in Table 1. Individual patient details are presented in Supplementary Tables 1 and 2 in Supplementary Information.

**Table 1:** Summary of clinical characteristics including age, initial tumour size, hormone receptor statuses, and cancer subtypes, of the studied LABC patients grouped by their clinical/pathological response to NACT

		Responders (*N* = 83)	Non-responders (*N* = 17)
**Age (yr)**	Min	31	29
	Max	83	67
	Mean	50	47
	SD	11	12
**Tumor size pre (cm)**	Min	1	3
	Max	12	13
	Mean	6	6
	SD	3	3
**ER positive**	No.	51	12
	%	61	71
**PR positive**	No.	45	11
	%	54	65
**HER2 positive**	No.	31	4
	%	37	24
**IDC**	No.	76	16
	%	92	94
**Other (ILC, IMC)**	No.	7	1
	%	8	6

Abbreviations: ER, estrogen receptor status; PR, progesterone receptor status; HER2, human epithelial growth factor receptor 2 status; IDC, invasive ductal carcinoma; ILC, invasive lobular carcinoma; IMC, invasive mammary carcinoma.

### Classification results

In this study, both two-class and three-class response grouping schemes were examined. In order to attain a sufficient number of samples for applying machine learning, the three-class response groups were combined into several two-class groups in the following manner: complete response (CR) versus (partial response (PR) + non-response (NR3)); (CR + PR) versus NR3; and PR versus NR3. Please refer to Materials and Methods for the definition of response types. Random down-sampling was performed on the majority class in order obtain a balanced set. Table 2 presents the majority and minority class sizes, balanced set size, and the number of balanced sets obtained after down-sampling for each grouping scheme. As observed, the number of balanced sets varies between grouping schemes depending on the number of times random sampling was required to sample all patients in the database.

**Table 2:** Majority and minority class sizes, balanced set size, and number of balanced sets used for each classification type, including responder (R) vs non-responder (NR2), complete responder (CR) + partial responder (PR) vs NR3, CR vs (PR+NR3), PR vs NR3, and survivor vs non-survivor

	Majority class	Minority class	Balanced set size	No. of balanced sets
**R vs NR2**	R (83)	NR2 (17)	34 (17+17)	21
**CR vs (PR+NR3)**	PR+NR3 (55)	CR (45)	90 (45+45)	5
**(CR+PR) vs NR3**	CR+PR (92)	NR3 (8)	16 (8+8)	41
**PR vs NR3**	PR (47)	NR3 (8)	16 (8+8)	22
**Survival**	Survived (86)	Not survived (14)	28 (14+14)	27

**Figure 1:**
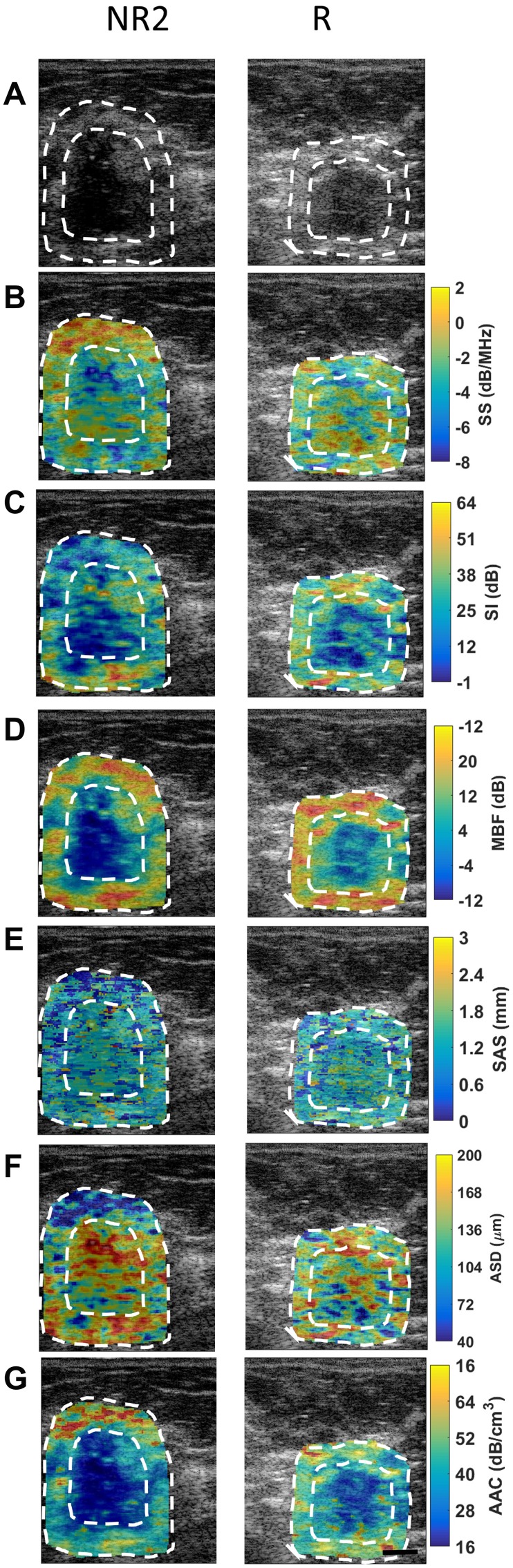
QUS images from a representative non-responder (NR2) patient and a representative responder patient with outlines of core and margin ROIs. (**A**) B-mode images, (**B**) SS image, (**C**) SI images, (**D**) MBF images, (**E**) SAS images, (**F**) ASD, and (**G**) AAC images obtained prior to chemotherapy treatment initiation. Scale bars: 1 cm.

**Figure 2:**
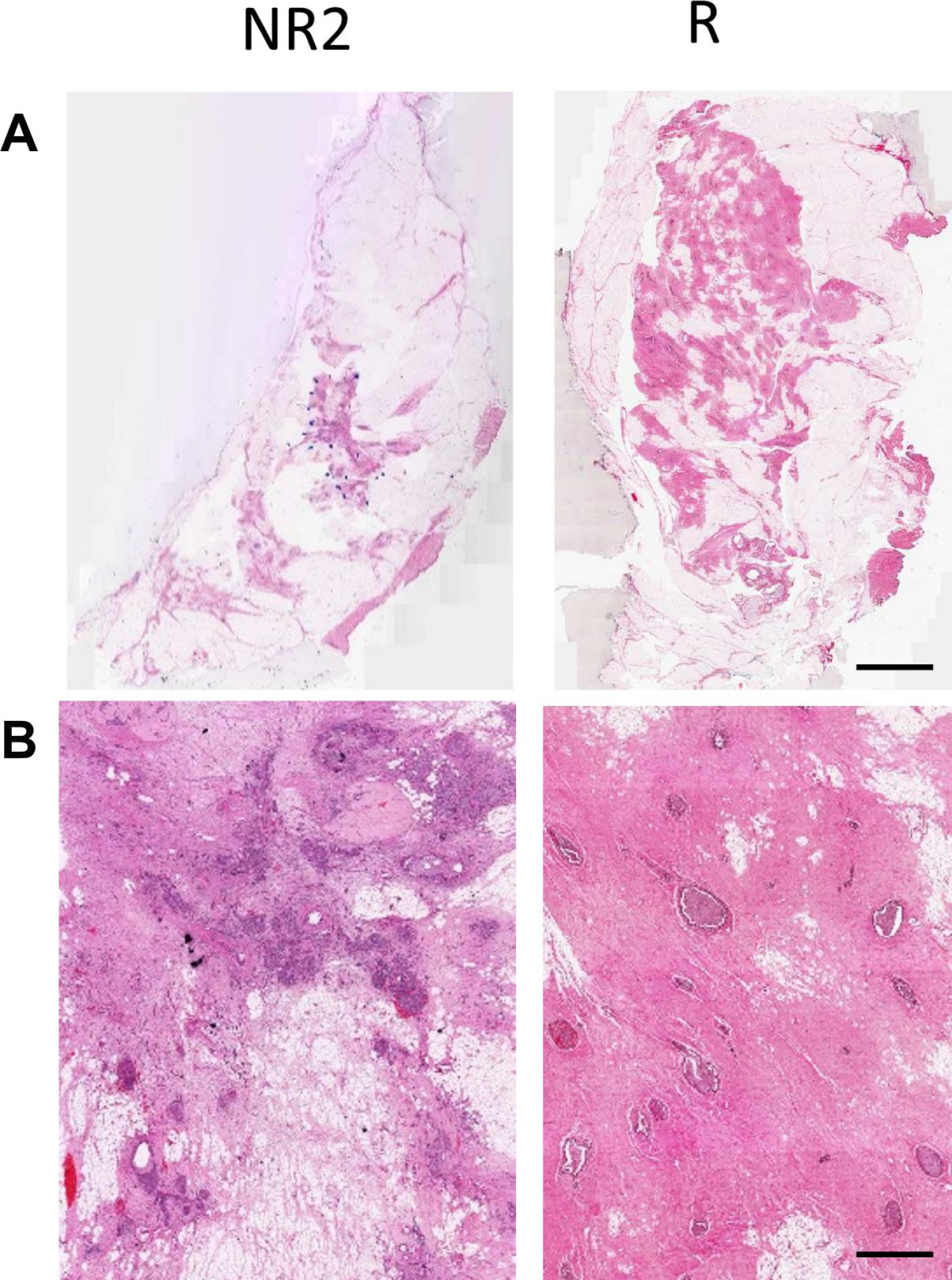
(**A**) H & E stained histology images of the excised breast specimen after resection. (**B**) High-magnification images. Scale bars: H & E low magnification – 1 cm, H & E high magnification – 100 μm.

Figure 1 presents representative responder and non-responder patient QUS images with outlines of the core and margin ROIs. Displayed are images of B-mode (A), SS (B), SI (C), MBF (D), SAS(E), ASD (F), and AAC (G) parametric maps of the patient’s tumour prior to treatment initiation. In Figure 2, the corresponding low-magnification (A) and high magnification (B) images of hematoxylin and eosin (H&E) stained sections of breast tissue specimens (excised after treatment completion and surgery) are displayed. It is evident from this figure that there are spatial variations in pixel intensities of the parametric images, highlighting the importance of texture-based features when discriminating responding tumours from non-responding ones.

**Figure 3:**
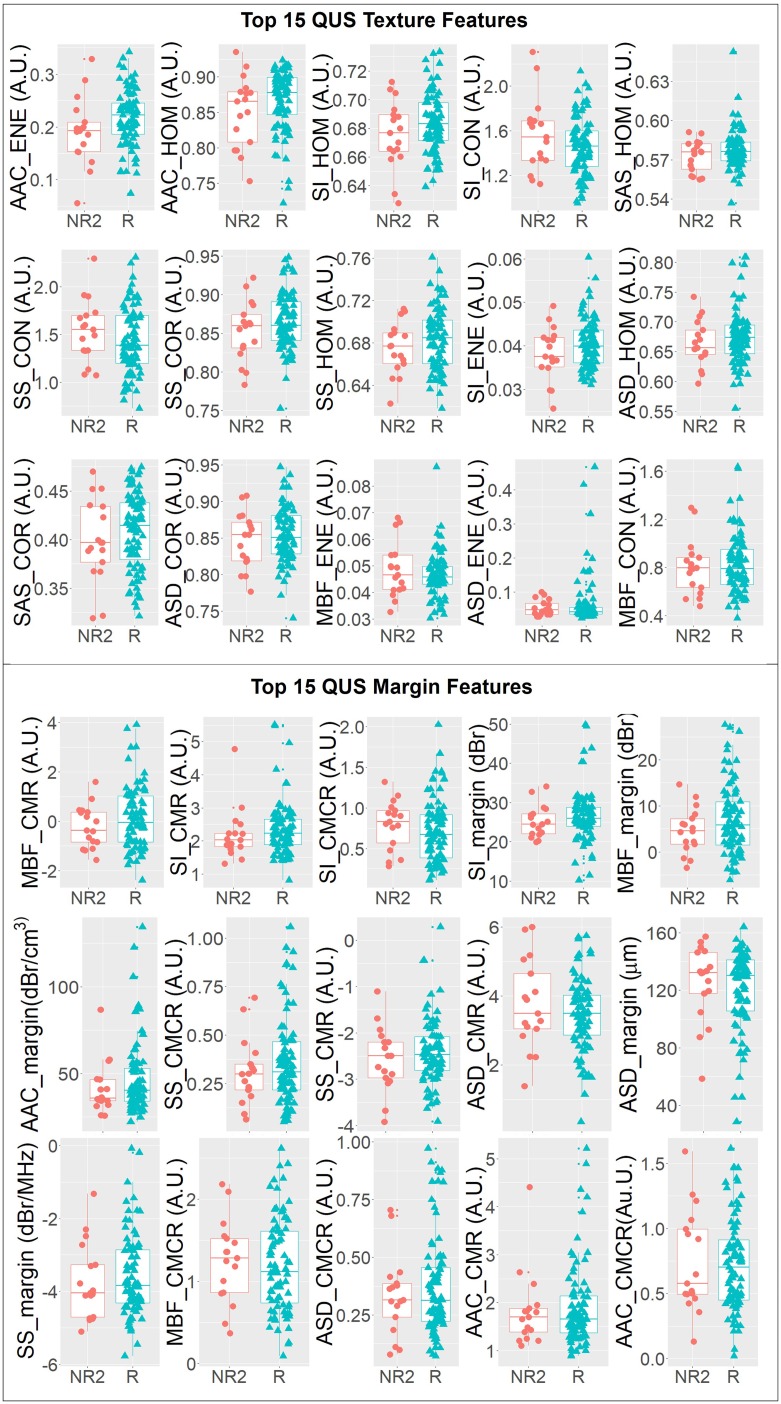
One-dimensional scatter plots and overlaid boxplots of the top 15 QUS texture featuers and top 15 QUS margin features comparing responder (R) and non-responder (NR2) groups. The features are plotted in order of statistical significance (smallest *p*-value to largest *p*-value) from left to right, top to bottom in a raster fasion.

Figure 3 compares responding and non-responding patients through a panel of overlaid scatter plots and box plots of the top 15 QUS texture features and the top 15 QUS margin features in order of statistical significance (*t*-test or Mann–Whitney test). None of the features plotted were found to be statistically significant on their own (*p* > 0.05). However, one parameter, AAC energy, was found to be marginally significant (*p* = 0.05). As evident from Figure 3, none of the individual QUS features are linearly separable between the responder and non-responder groups. This highlights the need for multi-feature classification and non-linear classifiers in order to solve this complex classification problem. Table 3 presents mean classification performance metrics obtained from running the ANN model on all balanced sets (the number of balanced sets varied from 5 to 41 depending on class distribution as reported in Table 2). Reported metrics include sensitivity, specificity, accuracy, and AUC evaluated on the test set. For conventional response (R) versus non-response (NR2) classification, mean values of sensitivity, specificity, accuracy, and AUC of 89 ± 9 %, 85 ± 12 %, 87 ± 6 %, and 0.90 ± 0.07 were obtained, respectively. In a three-class grouping scheme, when CR and PR patients were combined into one group and were compared against the NR3 patients, a 9% higher classification accuracy was observed on average (over the samples) compared to the conventional grouping scheme. This permitted non-responder patients and patients with response (partial or complete) to be identified up-front with an accuracy of 96 ± 6%. However, when CR patients were compared against PR+NR3 patients, the classification accuracy dropped by 8% compared to the conventional grouping scheme. Classification of PR versus NR3 patients yielded an accuracy of 86 ± 10 %. However, due to the relatively small sample size (47 PR and 8 NR3), the model has limited statistical power compared to the other classification types.

**Table 3:** Comparison of classification performances by ANN for different types of patient classification

		Sensitivity (%)	Specificity (%)	Accuracy (%)	AUC
**R vs NR2**	Mean	89	85	87	0.90
	SD	9	12	6	0.07
**CR vs (PR+NR3)**	Mean	83	75	79	0.79
	SD	3	1	1	0.04
**(CR+PR) vs NR3**	Mean	93	98	96	0.96
	SD	9	6	6	0.08
**PR vs NR3**	Mean	88	84	86	0.89
	SD	12	16	10	0.11
**Survival**	Mean	89	84	85	0.89
	SD	8	11	7	0.11

Reported values are mean and standard deviation (SD) values obtained by averaging the results over the subsets.

Classification performance for survival was also evaluated. The classification performance measures for classifying 5-year survivors versus patients with recurrence were similar to those for the conventional response classification (sensitivity, specificity, accuracy, and AUC of 89 ± 8 %, 84 ± 11 %, 85 ± 7 %, and 0.89 ± 0.11, respectively).

**Figure 4:**
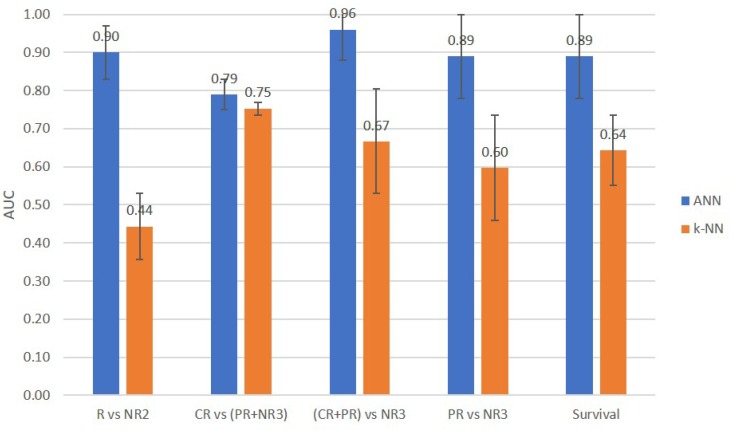
Comparison of prediction performance AUCs of the ANN and KNN classifiers for two-class and three-class response and survival prediction tasks.

Figure 4 compares the AUCs obtained using the ANN and KNN classifiers for predicting two-class and three-class responses and survival of patients. In classification tasks, the ANN classifier outperformed the KNN classifier. Table 4 presents, for each grouping scheme, the five QUS and hormone features selected by the sequential forward feature selection method that yielded the highest AUC. As evident, QUS texture features contributed prominently to the response prediction models in all grouping schemes. Hormone features did not contribute to the binary classification (conventional response prediction and survival prediction), whereas the opposite was true when patients were grouped based on their three-category response criteria: for CR vs (PR+NR3) classification, ERe and PRe contributed to the prediction; and for PR vs NR3 prediction, ERe contributed to the prediction.

**Table 4:** The five best QUS + molecular features obtained by the ANN classifier for different types of classification

	Best Features				
**R vs NR2**	AAC_ENE_IN_	AAC_HOM_IN_	MBF_CMR_	SI_HOM_IN_	SAS_ENE_IN_
**CR vs (PR+NR3)**	ER	SAS_ENE_IN_	SAS_CON_IN_	PR	MBF_HOM_IN_
**(CR+PR) vs NR3**	SS_ENE_IN_	SAS_HOM_IN_	AAC_ENE_IN_	ASD_HOM_IN_	ASD_COR_IN_
**PR vs NR3**	SAS_HOM_IN_	SAS_ENE_IN_	SAS_CON_IN_	SI_COR_IN_	ER
**Survival**	AAC_CON_IN_	ASD_CON_IN_	ASD_CORE_IN_	ASD_MARGIN_	SI_ENE_IN_

Reported are the five QUS + molecular features that were determined by way of cross-validation to yield the highest AUC.

## DISCUSSION

In this study, the statistical features of QUS images combined with an artificial neural network classifier were demonstrated, for the first time, to be effective in the pre-treatment prediction of response and 5-year recurrence-free survival of LABC patients receiving neoadjuvant chemotherapy. Both the conventional clinical response and recurrence-free survival were predicted with high accuracies (87 % and 85 % on average, respectively). Importantly, the best results were obtained when differentiating patients with no response versus those with some response ((CR+PR) versus NR3) with an accuracy of 96% on average (93 % sensitivity, 98% specificity and 0.96 AUC). The classification results were validated with patient modified response scores determined using post-surgical pathology data. The method proposed here can be incorporated, as a pre-treatment screening step, in the clinical workflow of LABC patients. This step would provide insight into the effectiveness of a given treatment regimen and allow the personalization of treatment. If it is known up-front that a patient will not respond to a particular chemotherapy, other agents or treatments can be selected instead of embarking on a several-month course of ineffective chemotherapy.

In this study, the ANN provided the best classification results. Results obtained using a KNN classifier were worse but were limited to 5 input parameters to avoid overfitting, whereas our previous work used more than 10. The high accuracy attained here is important for such methods to be used clinically. This can potentially lead to an improvement in patient quality of life as well as substantial savings in time, costs, and resources for both the patient and the health care provider.

The results demonstrated that the gray-level co-occurrence matrix (GLCM)-based texture features contributed to both response prediction models (conventional and three-class). The sensitivity of QUS texture features to therapy responsiveness are likely linked to the heterogeneous nature of tumour response to chemotherapy at the early stages. This theory has been suggested in previous studies examining GLCM-based [25] and local binary pattern-based [34] QUS texture analyses of LABC tumours undergoing chemotherapy. Aside from treatment response characterization, a previous LABC tumour characterization study demonstrated that QUS texture features provide a strong discrimination between low grade and medium-to-high grade tumours. [19], suggesting a link between QUS texture features and tumour heterogeneity.

Our response prediction results highlighted the sensitivity of the QUS feature set, identified by the ANN classifier, to the labels used in the training data set (Table 4). This may be due, in part, to the small number of non-responding patients (*N* = 17) compared to responding patients (*N* = 83). As data from new non-responding patients is collected in the future, the inter-patient variations in QUS features will be more effectively accounted for through machine learning and a more robust set of QUS features will be identified. The partial correlations between QUS features here are acknowledged. In parameters calculated through linear regression of the RF power spectrum, SS is related to the size of diffuse scatterers, SI is related to the acoustic concentration, and MBF is related to SS and SI. Among parameters using the Gaussian form factor model, ASD is an estimate of scatterer size and AAC is an estimate of acoustic concentration. However, due to the difference in the underlying models and assumptions, ASD and SS are partially correlated, and (MBF, SI) and AAC are partially correlated. In terms of ASD versus SAS, ASD characterizes the size of diffuse scatterers whereas SAS measures the spacing between both regular and diffuse scatterers. In a study characterizing diffuse liver disease, SAS measurements have been correlated with the distribution of collagen fibers [35]. In breast studies, ASD measurements have been correlated with the size of cells [17, 18]. Thus, it is plausible for SAS measurements in this study to be correlated with collagen fibers and cells, whereas ASD measurements are correlated with the distribution of cells.

There is mounting evidence suggesting that molecular subtypes (i.e. hormone receptor expressions) of tumours play an important role in developed or inherent drug resistance [36]. The fact that ERe and PRe contributed to the CR vs (PR+NR3) differentiation and that ER status was determined as a contributing parameter to PR vs NR3 differentiation confirmed the importance of tumour hormone receptor expression as a predictive marker. This has also been suggested in previous studies [6, 7]. Here, a hybrid model consisting of image-based and molecular-based markers was constructed employing an ANN classifier, which yielded similar accuracy to that of our previous work [32]. The current study includes two improvements: the patient cohort investigated is nearly double the size of cohort in the previous study, and data imbalance correction was made prior to classification through random sub-sampling. Furthermore, in this study, a survival predictor model was developed using an ANN classifier. In the previous study [32], retrospective survival analysis was performed (Kaplan-Meier survival curves), providing predictive insight into patient survival. In addition, various three-way classifiers resulted in better results for what is an important clinical indicator- identifying patients *a priori* who will have no response to chemotherapy.

The method developed here may also be combined with monitoring of cell-death responses using quantitative ultrasound. Sadeghi-Naini *et al* [25] have recently used similar approaches to monitor treatment response based on cell death detection using quantitative ultrasound. They indicated that QUS markers of response to NAC capture microstructural changes in the tumour induced by anticancer drugs, which correlate very well with long-term outcomes. Thus, it is not surprising that such markers could provide insight into the likelihood of response prior to starting treatment.

Previous studies have investigated methods for *a priori* prediction of treatment response. Tran *et al.* [37] recently demonstrated the utility of diffuse optical spectroscopy imaging, particularly the homogeneity texture feature of oxygenated haemoglobin concentration within the breast in predicting breast tumour response to NAC with an accuracy of 88%. However, that study was limited to a smaller analysis of 37 patients, nearly a third of the size of the cohort used here. Furthermore, uncertainties in tumour delineation arose due to the relatively low resolution of DOS.

Molecular markers have also been used to predict breast cancer recurrence. A 21-gene reverse transcriptase-polymerase chain reaction (RT-PCR) assay, or Oncotype DX [38], has been used to grade a recurrence risk of breast cancer in patients with lymph node negative, estrogen receptor-positive breast cancer. The recurrence score was found to be predictive of whether or not a patient would benefit from adjuvant chemotherapy. However, for now that technique applies only to the aforementioned sub-group of breast cancer patients, whereas the QUS method proposed here applies to all LABC patients. Furthermore, our method is potentially extendable to early breast cancer patients receiving up-front chemotherapy or adjuvant chemotherapy, regardless of their lymph node or hormone receptor statuses.

Drug resistance of cancer cells to chemotherapy can be inherent or developed through exposure to the drug [36]. A large body of research has established multidrug resistance (MDR) transporter proteins as one of the key mechanisms of cancer cell resistance to chemotherapy drugs [36], particularly anthracyclines and taxanes. Thus, as a future investigation, correlating QUS properties of a tumour with its MDR biomarkers may shed light on the mechanism by which QUS could detect inherent MDR in a tumour. As mentioned previously, Ki-67 is also an important pre-treatment biomarker of tumour responsiveness [4]. As Ki-67 is a cell proliferation biomarker that is present in the active phases of the cell cycle (G1, S, G2, and mitosis), it is a marker of cellular and glandular morphology. QUS- based tissue characterization works by discriminating tissues based on differences in their microstructure. In the 1-10 MHz range of frequencies used in clinical applications, ultrasound is sensitive to scatterers in the range of 20-500 μm in diameter [16]. Thus, it is plausible that ultrasound is sensitive to differences in the glandular morphology of tumours, which ultimately determines the likelihood of chemotherapy response. Most likely cellular changes associated with malignancy have an effect at one level, and as tumours become more aggressive the organization of cells becomes more and more deranged at the ductal level and then at the glandular level.

Due to the highly heterogeneous nature of tumours, particularly those of the breast, the prediction of their response to NAC requires advanced machine learning algorithms that can effectively learn a non-linear pattern from data and build a strong classifier from several weak classifiers (QUS & molecular features). One of the most popular machine learning techniques with this capability is artificial neural networks. Recently, artificial neural networks have gained interest in oncology through successful applications in the detection of breast cancer in mammography images (AUC of 0.82) [39] and in the detection and localization of cancer metastasis in whole-slide pathology images of lymph nodes (AUC above 0.97) [40].

In the study here, an image-based model including textural features and tumour/periphery analyses was proposed for predicting response to NAC and survival of LABC patients. The sonographic analyses here can be thought of as generating “sonomic” biomarkers of response prediction akin to genomic biomarkers for predictive or prognostic assays but derived through ultrasound analyses as opposed to genetic analyses. Pre-treatment image-based biomarker surrogates of response stand to personalize health care by minimizing drug toxicity and maximizing chances of long-term survival. The technology can be incorporated into existing commercial clinical ultrasound imaging systems capable of RF data acquisition and potentially extended to other cancer types.

## MATERIALS AND METHODS

This prospective study was reviewed and approved by the institution’s research ethics board. After obtaining informed consent, ultrasound RF data were acquired from 100 patients with biopsy-confirmed LABC prior to start of their NAC. Data acquisition was performed by an experienced sonographer using a Sonix RP system (Ultrasonix, Vancouver, Canada) equipped with a 6 MHz linear array transducer (L14-5/60W) with a digital sampling rate of 40 MHz. The focus was set at the midline of the tumour using electronic beam focusing, and the imaging depth ranged from 4 to 6 cm, depending on tumour size and location. Images were acquired at 5 mm intervals over the tumour volume.

### Patient clinical characteristics

Patient data including age, initial tumour size (measured by imaging), ERe status, PRe status, and HER2 status were recorded. The clinical/pathological tumour response of each patient to treatment was determined at the end of their treatment using a modified response (MR) grading system which was based on RECIST [41] and histological [42] criteria. The MR score was defined as follows: MR Score 1: no diminishment in tumour size (cNR); MR2: up to 30% diminishment in tumour size (cNR); MR 3: between an estimated 30% and 90% reduction in tumour size (cPR); MR 4: a diminishment of more than 90% in tumour size (almost pCR); MR 5: no evident tumour and no malignant cells identifiable in sections from the site of the tumour; only vascular fibroelastotic stroma remains, often containing macrophages; however, ductal carcinoma *in situ* may be present (pCR).

Both binary and three-class classifications were investigated. In the binary scenario, a patient with an MR score of 3-5 was deemed to be a responder (R) and a patient with an MR score of 1–2 was deemed to be a non-responder (NR2). In the three-class scenario, a patient with MR grade of 4–5 was deemed to be a complete responder (CR), 2–3 a partial responder (PR), and 1 a non-responder (NR3). The number proceeding NR (i.e. NR2 or NR3) differentiates the non-responders in the two-class and three-class grouping schemes. All patients received anthracycline/taxane-based treatment lasting several months. Each patient received a treatment regimen according to their disease type, stage, and hormone receptor expressions. Details about the specific types of treatments administered to individual patients are provided in Supplementary Table 1 in Supplementary Information. Recurrence-free survival was determined based on a 5-year follow up timeframe, during which the patient was free of any local or distant cancer recurrence.

### QUS feature evaluation

QUS analysis was carried out using the dual ROI method published previously [32]. In each B-mode breast ultrasound image, two separate ROIs consisting of 1) tumour core and 2) a rim of surrounding tissue of 5 mm thickness were manually contoured. This process was repeated on 4-7 image planes across the tumour. All images were para-sagittal. All images were non-overlapping. For each ROI, QUS features were computed within sliding RF windows that were 2 × 2 mm in size and had 94% overlap in axial and lateral directions to produce a parametric map. From each parametric map, mean of intensity, texture, and image quality metrics were extracted and averaged across the image planes and subsequently used as features (inputs) for the ANN-based patient response classifier. The QUS parametric maps that were generated from the raw ultrasound data were: MBF, SS, SI, ASD, AAC, SAS, and attenuation coefficient estimate (ACE). In order to characterize structural patterns in the parametric maps, a gray-level co-occurrence matrix (GLCM)–based texture analysis was performed on the newly obtained parametric images as described in Tadayyon *et al*. [19]. This method was originally developed by Haralick *et al*. [43]. The texture features that were extracted from the parametric maps were contrast (CON), correlation (COR), energy (ENE), and homogeneity (HOM). Additionally, two image quality metrics were extracted from the parametric maps that compared the statistical properties of the core ROI to those of the margin ROI: core-to-margin ratio (CMR), and core-to-margin contrast ratio (CMCR) as per the method described in [32]. Table 5 presents, in detail, the QUS-based features that were included in the ANN-based response classifier model based on ROI location and image metric. In addition to above-mentioned QUS features, tumour receptor expression statuses including PRe, ERe, an HER2 were investigated in the analysis. In total, 52 features were investigated as potential predictors of response. Details about the QUS analysis are provided in Supplementary Information.

**Table 5:** QUS image-based features that were computed per patient, identified by ROI (core or margin) and image metric (mean, texture, CMR, or CMCR)

	Core ROI	Margin ROI	Core vs. Margin	no. of features
**Mean**	MBF, SS, SI, SAS, ASD, AAC, ACE	MBF, SS, SI, SAS, ASD, AAC		13
**Texture (CON, COR, ENE, HOM)**	MBF, SS, SI, SAS, ASD, AAC,			24
**CMR**			MBF, SS, SI, SAS, ASD, AAC,	6
**CMCR**			MBF, SS, SI, SAS, ASD, AAC,	6
**TOTAL**				49

A total of 49 features were computed per patient.

### Response and recurrence-free survival classification

Prior to applying a classification rule, a data balancing step was performed by way of down-sampling to account for the smaller sample size of non-responding patients (MR=1-2, N=17) compared to responding patients (MR=3-5, N=83). In this step, random samples (with replacement) were drawn from the majority class QUS data (responding patients) with a size equal to that of the minority class (non-responding patients). This was repeated as many times as required to sample all patients in the majority class. Sequential forward feature selection (SFFS) [44] with *p*-value initialization was applied to the balanced dataset to determine the optimal feature set for classification. This involved sorting the features based on their *p*-values of significance (from smallest to largest) obtained from an unpaired two-sample *t*-test or Mann–Whitney test, starting with the first feature as the initial feature set and adding or discarding features using the SFFS method until all features were evaluated. The maximum feature size was set to 5 in order to avoid overfitting due to the high dimensionality of the data set. The ANN classifier was configured as a single hidden layer model. In each balanced set, the data was randomly split into 70% training, 15% validation, and 15% test sets. In the training phase, hyper-parameter tuning was performed on the hidden layer size (1–10 nodes) using the training set to train the network and the validation set to evaluate it based on AUC. The test set was used to evaluate the generalization error of the network after fixing its hyper-parameters. The process was repeated 10 times on 10 bootstrapped, train-validate-test sets in order to account for variations in the ANN output due to random sampling. For each balanced set, a verification step was conducted to ensure that there was at least one sample from each class in each of the training, validation, and testing sets. For each balanced set, classifier performance metrics including sensitivity, specificity, accuracy, and AUC were measured on the test sets, which were obtained by averaging the values over the 10 bootstrap samples. For comparison, a KNN model was trained and tested in the same manner. Sensitivity was defined as the ratio of the number of true positives (responders) to the total number of positives in the test set. Specificity was defined as the ratio of the number of true negatives (non-responders) to the total number of negatives in the test set. Accuracy was defined as the ratio of the number of correctly classified patients to the total number of patients in the test set. All values are reported in percentages.

## SUPPLEMENTARY MATERIALS






