# Supplementary material for: A priori prediction of breast tumour response to chemotherapy using quantitative ultrasound imaging and artificial neural networks
Source: Oncotarget. 2019 Jun 11;10(39):3910–23. doi: 10.18632/oncotarget.26996 (PMC6570472; doi:10.18632/oncotarget.26996)
Supplement: Supplementary file 1 [file oncotarget-10-3910-s001.pdf]

A priori prediction of breast tumour response to chemotherapy using quantitative ultrasound imaging and artificial neural networks

SUPPLEMENTARY MATERIALS

QUS Feature evaluation

QUS Analysis was carried out using the dual ROI method published previously [1]. In each B-mode breast ultrasound image, tumour core and 5 mm surrounding tissue ROIs were manually contoured. Each ROI was divided into analysis blocks of 2 × 2 mm in size with 94% overlap in axial and lateral directions. This ensured a standardization of the QUS analysis block size among all patients. For each ROI, the mean normalized power spectrum was computed from each block via fast Fourier transform and used tissue-equivalent-phantom-based normalization, as described previously [2]. The normalized power spectrum was then subjected to attenuation correction and linear regression calculation and parameters including MBF, SS, and SI were determined. Attenuation correction was performed using the point-compensation method [3] after obtaining an estimate of the local attenuation coefficient (ACE) via the reference phantom technique [4].

For SAS estimation, the power spectrum of the tumour was estimated using the autoregressive (AR) model and the AR model parameters were estimated using Burg's recursive algorithm [5]. For SAS computation, the power spectrum was estimated using the AR-method rather than the FFT since the former offers two advantages – it produces more conspicuous peaks, resulting in more accurate estimates of SAS, and it is less prone to ringing artifacts at small gate lengths [6]. The order of the AR model, p, was determined experimentally using an ultrasound image of an LABC patient's breast. The value was chosen by plotting the spectral autocorrelations (SAC) for a range of p-values (from 10 to 100) and finding the *p*-value at which the peak in the SAC was most conspicuous and did not contain multiple peaks. This value was experimentally determined to be 50 for the breast ultrasound data that was used in this study. The power spectrum was then normalized to that of a planar reflector. The planar reflector normalization at different depths was performed using pre-recorded reference RF data acquired from a Plexiglas-water interface at six different depths (1–6 cm). For each RF block in the sample image, a reference RF block was selected by a nearest neighbour approach. By computing the autocorrelation of the normalized power spectrum, the SAS parameter was determined from the frequency at which the autocorrelation peak occurred. The method used here for SAS estimation is described elsewhere [7].

Next, using backscatter coefficient estimation and form factor model fitting [2], estimates of ASD and AAC were obtained. By generating a spatial map of the parameter values computed over all analysis blocks, parametric images were generated. In order to evaluate differences in spatial patterns of parametric images in the responding and non-responding groups, texture features were computed via a gray-level co-occurrence (GLCM) matrix calculation on the parametric images [8, 9]. The GLCM represents, statistically, the angular relationship between neighbouring pixels as well as the distance between them [8]. Texture features computed included contrast (CON), correlation (COR), energy (ENE), and homogeneity (HOM). Contrast gives a measure of intensity variation between pixel pairs over a ROI. Correlation provides a measure of intensity-level linear dependence between two pixels. Energy gives the squared sum of the elements in the GLCM. Homogeneity quantifies the closeness of the distribution of GLCM elements to the GLCM diagonal (the diagonal of the GLCM represents all pixel pairs with the same intensity). Sixteen symmetric GLCMs were constructed for each parametric map, corresponding to four pixel-to-pixel distances (1 pixel, 2 pixels, 3 pixels, and 4 pixels) and four directions (0°, 45°, 90°, and 135°). The texture features for each of the 16 symmetric GLCMs were computed and averaged to obtain a mean value. This resulted in a total of 24 texture features computed from the tumour core ROI since there were four texture features for each of the six parametric maps (MBF, SS, SI, SAS, ASD, and AAC; ACE was a single-value parameter). Texture features were not computed from the tumour margin ROI due to the size limitation of the ROI.

In addition to tumour core texture features, four additional features were computed from each of the six parametric maps: mean of intensity in the core ROI, mean of intensity in the margin ROI, core-to-margin ratio (CMR), and core-to-margin contrast ratio (CMCR). CMR was defined as the ratio of the mean of the core ROI to the standard deviation of the margin ROI, and CMCR was defined as the ratio of the difference between the mean of core ROI and mean of margin ROI to the mean of the standard deviations of the core and margin ROIs, as previously defined in [1]. These image metrics

are image quality features similar to signal-to-noise ratio (SNR) and contrast-to-noise ratio (CNR) typically used to assess image quality in medical imaging systems as X-ray computed tomography systems [10]. A total of 52 features were submitted to the ANN classifier for tumour response classification.

REFERENCES

1. Tadayyon H, Sannachi L, Gangeh MJ, Kim C, Ghandi S, Trudeau M, Pritchard K, Tran WT, Slodkowska E, Sadeghi-Naini A, Czarnota GJ. A priori Prediction of Neoadjuvant Chemotherapy Response and Survival in Breast Cancer Patients using Quantitative Ultrasound. Sci Rep. 2017; 7:45733. https://doi.org/10.1038/srep45733. [PubMed]
2. Tadayyon H, Sannachi L, Gangeh M, Sadeghi-Naini A, Tran W, Trudeau ME, Pritchard K, Ghandi S, Verma S, Czarnota GJ. Quantitative ultrasound assessment of breast tumor response to chemotherapy using a multi-parameter approach. Oncotarget. 2016; 7:45094–111. https://doi.org/10.18632/oncotarget.8862. [PubMed]
3. Oelze ML, O'Brien WD Jr. Frequency-dependent attenuation-compensation functions for ultrasonic signals backscattered from random media. J Acoust Soc Am. 2002; 111:2308–19. https://doi.org/10.1121/1.1452743. [PubMed]
4. Labyed Y, Bigelow TA. Estimating the total ultrasound attenuation along the propagation path by using a reference phantom. J Acoust Soc Am. 2010; 128:3232–38. https://doi.org/10.1121/1.3483739. [PubMed]
5. Wear KA, Wagner RF, Garra BS. High resolution ultrasonic backscatter coefficient estimation based on autoregressive spectral estimation using Burg's algorithm. IEEE Trans Med Imaging. 1994; 13:500–07. https://doi.org/10.1109/42.310881. [PubMed]
6. Wear KA, Wagner RF, Insana MF, Hall TJ. Application of autoregressive spectral analysis to cepstral estimation of mean scatterer spacing. IEEE Trans Ultrason Ferroelectr Freq Control. 1993; 40:50–8. https://doi.org/10.1109/58.184998. [PubMed]
7. Tadayyon H, Sadeghi-Naini A, Wirtzfeld L, Wright FC, Czarnota G. Quantitative ultrasound characterization of locally advanced breast cancer by estimation of its scatterer properties. Med Phys. 2014; 41:012903. https://doi.org/10.1118/1.4852875. [PubMed]
8. Haralick RM, Shanmugam K, Dinstein I. Textural Features for Image Classification. IEEE Trans. Syst. Man. Cybern.SMC. 1973; 3:610–21. https://doi.org/10.1109/TSMC.1973.4309314.
9. Tadayyon H, Sadeghi-Naini A, Czarnota GJ. Noninvasive characterization of locally advanced breast cancer using textural analysis of quantitative ultrasound parametric images. Transl Oncol. 2014; 7:759–67. https://doi.org/10.1016/j.tranon.2014.10.007. [PubMed]
10. Gayou O. Influence of acquisition parameters on MV-CBCT image quality. J Appl Clin Med Phys. 2012; 13:3638. https://doi.org/10.1120/jacmp.v13i1.3638. [PubMed]

Supplementary Table 1: Pre-treatment clinical characteristics of individual patient subjects. See Supplementary_Table_1

Supplementary Table 2: Treatment outcomes of individual patient subjects. See Supplementary_Table_2
