# Supplementary material for: A priori prediction of breast tumour response to chemotherapy using quantitative ultrasound imaging and artificial neural networks
Source: Oncotarget. 2019 Jun 11;10(39):3910–23. doi: 10.18632/oncotarget.26996 (PMC6570472; doi:10.18632/oncotarget.26996)
Supplement: Supplementary file 2 [file oncotarget-10-3910-s002.doc]

**Supplementary Table 1:** **Pre-treatment clinical characteristics of individual patient subjects**

| **Patient No.** | **Age** | **Tumor size (pre-tx)** | **Histology** | **ER** | **PR** | **HER2** | **Treatment** |
| --- | --- | --- | --- | --- | --- | --- | --- |
| 1 | 55 | 5.40 | IDC | - | - | + | FEC+D |
| 2 | 53 | 7.30 | IDC | + | + | - | EDP |
| 3 | 41 | 5.30 | IDC | + | + | + | TC |
| 4 | 50 | 4.60 | IDC | + | + | + | AC+D |
| 5 | 33 | 5.00 | ILC | + | + | - | AC+T |
| 6 | 33 | 8.00 | IDC | + | + | + | AC+T |
| 7 | 48 | 4.90 | IDC | + | + | - | AC+D |
| 8 | 36 | 5.80 | IDC | + | + | - | AC+D |
| 9 | 40 | 4.40 | IDC | - | - | - | AC+T |
| 10 | 62 | 10.00 | IDC | - | - | - | D |
| 11 | 59 | 6.00 | IDC | - | - | - | AC+T |
| 12 | 38 | 9.20 | IDC | + | + | - | AC+T |
| 13 | 48 | 4.30 | IDC | + | + | + | AC+T |
| 14 | 49 | 12.00 | IDC | - | - | + | AC+T |
| 15 | 46 | 7.00 | IDC | - | - | - | AC+T |
| 16 | 40 | 3.00 | IDC | - | + | + | AC+T |
| 17 | 56 | 3.20 | IDC | - | + | + | AC+T |
| 18 | 49 | 5.60 | IDC | - | - | + | AC+T |
| 19 | 47 | 5.20 | IDC | + | + | - | FEC+D |
| 20 | 52 | 4.10 | IDC | + | + | - | AC+T |
| 21 | 44 | 9.90 | IDC | + | + | + | AC+T |
| 22 | 38 | 9.00 | IDC | + | + | - | AC+T |
| 23 | 58 | 2.00 | IDC | - | - | - | AC+T |
| 24 | 39 | 12.00 | IDC | + | + | - | AC+T |
| 25 | 35 | 5.90 | IDC | - | - | - | AC+T |
| 26 | 38 | 2.60 | IDC | - | - | + | AC+T |
| 27 | 47 | 9.90 | IDC | + | + | - | AC+T |
| 28 | 57 | 5.50 | IDC | - | - | - | AC+T |
| 29 | 47 | 7.40 | IDC | - | - | + | AC+T |
| 30 | 55 | 12.80 | IDC | + | + | - | AC+T |
| 31 | 32 | 7.00 | IDC | + | + | + | AC+T |
| 32 | 38 | 2.50 | IDC | - | - | - | AC+T |
| 33 | 45 | 6.00 | IDC | + | + | + | AC+T |
| 34 | 55 | 10.50 | IDC | - | - | - | AC+T |
| 35 | 59 | 8.00 | IDC | + | - | + | FEC+D |
| 36 | 54 | 4.20 | IDC | + | + | - | FEC+D |
| 37 | 37 | 3.60 | IDC | + | + | - | AC+T |
| 38 | 50 | 9.00 | IDC | + | + | + | AC+T |
| 39 | 54 | 3.60 | IDC | + | + | - | TC |
| 40 | 55 | 1.60 | IMC | + | - | - | FEC+D |
| 41 | 50 | 7.30 | IDC | + | - | - | FEC+D |
| 42 | 55 | 3.40 | IDC | - | - | - | TC |
| 43 | 64 | 8.70 | ILC | + | + | - | FEC+D |
| 44 | 67 | 2.50 | IDC | - | - | - | FEC+D |
| 45 | 52 | 2.60 | IDC | - | - | - | FEC+D |
| 46 | 45 | 2.30 | IDC | + | + | + | FEC+D |
| 47 | 66 | 5.20 | IDC | + | + | + | TC |
| 48 | 49 | 2.10 | IDC | + | + | + | AC+T |
| 49 | 39 | 6.30 | IDC | + | + | - | FEC+D |
| 50 | 62 | 6.30 | IDC | - | - | - | AC+T |
| 51 | 58 | 5.20 | IDC | + | + | + | AC+T |
| 52 | 58 | 4.00 | IMC | - | - | + | TC |
| 53 | 45 | 4.00 | IDC | + | + | - | AC+T |
| 54 | 29 | 4.20 | IDC | + | + | - | AC+T |
| 55 | 79 | 3.90 | IDC | + | - | + | AC+T |
| 56 | 42 | 9.60 | IDC | + | + | - | FEC+D |
| 57 | 66 | 3.00 | IDC | + | - | + | FEC+D |
| 58 | 38 | 5.00 | IDC | - | - | - | AC+T |
| 59 | 40 | 11.70 | IDC | + | + | + | AC+T |
| 60 | 53 | 8.80 | IDC | - | - | - | FEC+D |
| 61 | 47 | 3.50 | IDC | + | + | - | AC+T |
| 62 | 57 | 3.90 | IDC | + | - | - | AC+T |
| 63 | 63 | 3.00 | IDC | + | + | - | FEC+D |
| 64 | 47 | 2.40 | IDC | - | - | - | AC+T |
| 65 | 41 | 7.90 | IDC | + | + | + | AC+T |
| 66 | 43 | 6.60 | IDC | + | + | - | AC+T |
| 67 | 38 | 4.80 | IDC | - | - | - | AC |
| 68 | 69 | 4.30 | IDC | + | + | - | FEC+D |
| 69 | 51 | 4.20 | IDC | - | - | + | AC+T |
| 70 | 53 | 5.60 | IDC | - | - | + | AC+T |
| 71 | 55 | 7.90 | IDC | + | + | + | FEC+D |
| 72 | 51 | 2.20 | IDC | + | + | - | AC+T |
| 73 | 55 | 3.10 | IDC | + | - | + | FEC+D |
| 74 | 31 | 4.00 | IDC | + | + | - | AC+T |
| 75 | 41 | 2.20 | ILC | + | + | - | AC+T |
| 76 | 53 | 2.30 | IDC | + | + | - | FEC+D |
| 77 | 42 | 3.10 | IDC | - | - | - | AC+T |
| 78 | 31 | 3.50 | IDC | - | - | - | AC+T |
| 79 | 32 | 5.60 | IDC | - | - | - | AC+T |
| 80 | 45 | 8.10 | IDC | - | - | + | TC |
| 81 | 53 | 5.30 | IDC | - | - | - | FEC+D |
| 82 | 58 | 5.30 | IDC | + | + | + | FEC+D |
| 83 | 38 | 10.90 | IDC | + | + | - | FEC+D |
| 84 | 72 | 3.30 | IDC | + | - | - | AC+T |
| 85 | 41 | 4.50 | IDC | - | - | - | AC+T |
| 86 | 48 | 5.60 | ILC | + | + | - | FEC+D |
| 87 | 83 | 7.70 | IDC | + | - | - | AC+T |
| 88 | 51 | 4.50 | IDC | + | + | - | AC+T |
| 89 | 43 | 9.00 | IDC | - | - | - | AC+T |
| 90 | 42 | 5.00 | IDC | - | - | - | FEC+D |
| 91 | 60 | 7.20 | IDC | + | + | + | AC+T |
| 92 | 42 | 6.30 | IDC | + | + | - | FEC+D |
| 93 | 47 | 10.40 | IDC | + | + | - | AC+T |
| 94 | 45 | 7.30 | IDC | + | + | - | FEC+D |
| 95 | 43 | 8.50 | IMC | - | - | - | FEC+D |
| 96 | 62 | 2.10 | IAC | - | - | - | CT |
| 97 | 42 | 6.00 | IDC | + | + | + | FEC+D |
| 98 | 72 | 1.25 | IDC | + | + | + | AC+T |
| 99 | 63 | 8.60 | IDC | + | + | - | AC+T |
| 100 | 68 | 2.20 | IDC | + | + | + | AC+T |

Presented are age, estrogen receptor (ER), progesterone receptor (PR), and human epithelial growth factor 2 (HER2) tumor statuses, tumor histological subtype (IDC = invasive ducal carcinoma, ILC = invasive lobular carcinoma, IMC = invasive mammary carcinoma), pre-treatment tumor size (longest dimeS1ion), source of the size measurement, and treatment regimen (A = anthracycline, T = taxol, FEC = Fluorouracil-Epirubicin-Cyclophosphamide, D = Docetaxel, AC = Adriamycin and cyclophosphamide, EDP = etoposide, doxorubicin, cisplantium, TC = taxotere and cyclophosphamide).
