# Supplementary material for: A priori prediction of breast tumour response to chemotherapy using quantitative ultrasound imaging and artificial neural networks
Source: Oncotarget. 2019 Jun 11;10(39):3910–23. doi: 10.18632/oncotarget.26996 (PMC6570472; doi:10.18632/oncotarget.26996)
Supplement: Supplementary file 3 [file oncotarget-10-3910-s003.doc]

**Supplementary Table 2:** **Treatment outcomes of individual patient subjects**

| **Patient No.** | **Tumor size (post-tx)** | **Miller-Payne grade** | **R vs NR2** | **CR vs (NR+PR)** | **(CR+PR) vs NR** | **Survival** |
| --- | --- | --- | --- | --- | --- | --- |
| 1 | 0.00 | 5 | + | + | + | + |
| 2 | 7.00 | 3 | + | - | + | + |
| 3 | 2.70 | 2 | - | - | + | - |
| 4 | 0.00 | 5 | + | + | + | + |
| 5 | 1.40 | 3 | + | - | + | + |
| 6 | 0.00 | 5 | + | + | + | + |
| 7 | 1.40 | 3 | + | - | + | + |
| 8 | 11.40 | 1 | - | - | - | - |
| 9 | 2.00 | 5 | + | + | + | + |
| 10 | 0.00 | 5 | + | + | + | + |
| 11 | 2.60 | 3 | + | - | + | - |
| 12 | 4.50 | 3 | + | - | + | + |
| 13 | 5.00 | 3 | + | - | + | + |
| 14 | 2.00 | 5 | + | + | + | + |
| 15 | 0.00 | 5 | + | + | + | + |
| 16 | 0.00 | 5 | + | + | + | + |
| 17 | 0.20 | 4 | + | + | + | + |
| 18 | 0.10 | 4 | + | + | + | + |
| 19 | 6.50 | 4 | + | + | + | + |
| 20 | 0.00 | 5 | + | + | + | + |
| 21 | 2.00 | 4 | + | + | + | + |
| 22 | 2.00 | 3 | + | - | + | - |
| 23 | 0.20 | 5 | + | + | + | + |
| 24 | 8.00 | 3 | + | - | + | + |
| 25 | 0.00 | 5 | + | + | + | + |
| 26 | 0.00 | 5 | + | + | + | + |
| 27 | 18.00 | 3 | + | - | + | - |
| 28 | 0.00 | 5 | + | + | + | + |
| 29 | 0.00 | 5 | + | + | + | + |
| 30 | 17.00 | 1 | - | - | - | - |
| 31 | 7.40 | 2 | - | - | + | + |
| 32 | 3.80 | 1 | - | - | - | + |
| 33 | 4.80 | 2 | - | - | + | - |
| 34 | 0.10 | 4 | + | + | + | + |
| 35 | 0.00 | 5 | + | + | + | + |
| 36 | 4.00 | 2 | - | - | + | + |
| 37 | 2.20 | 3 | + | - | + | - |
| 38 | 1.20 | 3 | + | - | + | - |
| 39 | 1.70 | 3 | + | - | + | + |
| 40 | 1.20 | 3 | + | - | + | + |
| 41 | 2.10 | 3 | + | - | + | + |
| 42 | 1.80 | 3 | + | - | + | + |
| 43 | 19.00 | 1 | - | - | - | - |
| 44 | 3.20 | 1 | - | - | - | + |
| 45 | 2.40 | 3 | + | - | + | + |
| 46 | 0.00 | 5 | + | + | + | + |
| 47 | 4.00 | 3 | + | - | + | + |
| 48 | 1.60 | 5 | + | + | + | + |
| 49 | 1.70 | 3 | + | - | + | + |
| 50 | 12.60 | 1 | - | - | - | - |
| 51 | 3.40 | 3 | + | - | + | + |
| 52 | 0.00 | 5 | + | + | + | + |
| 53 | 3.00 | 3 | + | - | + | + |
| 54 | 4.00 | 2 | - | - | + | + |
| 55 | <1 | 3 | + | - | + | - |
| 56 | 3.00 | 4 | + | + | + | + |
| 57 | 2.40 | 2 | - | - | + | + |
| 58 | 5.00 | 2 | - | - | + | + |
| 59 | 1.30 | 3 | + | - | + | + |
| 60 | 2.50 | 3 | + | - | + | - |
| 61 | 4.00 | 3 | + | - | + | + |
| 62 | 3.30 | 3 | + | - | + | + |
| 63 | 0.50 | 3 | + | - | + | + |
| 64 | 0.00 | 5 | + | + | + | + |
| 65 | 0.20 | 4 | + | + | + | + |
| 66 | 4.00 | 3 | + | - | + | + |
| 67 | 0.00 | 5 | + | + | + | + |
| 68 | 2.00 | 3 | + | - | + | + |
| 69 | 0.00 | 5 | + | + | + | + |
| 70 | 0.20 | 4 | + | + | + | + |
| 71 | 12.60 | 3 | + | - | + | + |
| 72 | 0.50 | 3 | + | - | + | + |
| 73 | 0.00 | 5 | + | + | + | + |
| 74 | 1.70 | 3 | + | - | + | + |
| 75 | 1.10 | 4 | + | + | + | + |
| 76 | 0.00 | 5 | + | + | + | + |
| 77 | 0.00 | 5 | + | + | + | + |
| 78 | 0.00 | 5 | + | + | + | + |
| 79 | 2.70 | 3 | + | - | + | - |
| 80 | 1.60 | 4 | + | + | + | + |
| 81 | 0.00 | 5 | + | + | + | + |
| 82 | 1.50 | 4 | + | + | + | + |
| 83 | 4.90 | 2 | - | - | + | + |
| 84 | 0.20 | 4 | + | + | + | + |
| 85 | 2.00 | 3 | + | - | + | + |
| 86 | 11.00 | 3 | + | - | + | + |
| 87 | 0.00 | 5 | + | + | + | + |
| 88 | 3.50 | 2 | - | - | + | + |
| 89 | 3.90 | 3 | + | - | + | + |
| 90 | 8.00 | 1 | - | - | - | + |
| 91 | 1.00 | 4 | + | + | + | + |
| 92 | 8.40 | 1 | - | - | - | + |
| 93 | 4.50 | 4 | + | + | + | + |
| 94 | 1.40 | 3 | + | - | + | + |
| 95 | 0.01 | 4 | + | + | + | + |
| 96 | 0.00 | 5 | + | + | + | + |
| 97 | 2.50 | 3 | + | - | + | + |
| 98 | 0.00 | 5 | + | + | + | + |
| 99 | 7.50 | 3 | + | - | + | + |
| 100 | 0.00 | 5 | + | + | + | + |

Presented are post-treatment tumor bed size in cm (note that 0 indicates complete disappearance of the tumor mass), MP score (1-5), and different groupings of response based on the MP grade. Additionally, the survival outcome at 5 years is indicated.
